# Interplay between Microbiota and γδ T Cells: Insights into Immune Homeostasis and Neuro-Immune Interactions

**DOI:** 10.3390/ijms25031747

**Published:** 2024-02-01

**Authors:** Alaa A. Mohamed, Basel K. al-Ramadi, Maria J. Fernandez-Cabezudo

**Affiliations:** 1 Department of Biochemistry and Molecular Biology, College of Medicine and Health Sciences, United Arab Emirates University, Al-Ain P.O. Box 15551, United Arab Emirates; 2 Department of Medical Microbiology and Immunology, College of Medicine and Health Sciences, United Arab Emirates University, Al-Ain P.O. Box 15551, United Arab Emirates; 3 Zayed Center for Health Sciences, United Arab Emirates University, Al Ain P.O. Box 15551, United Arab Emirates

**Keywords:** microbiota, inflammation, γδ T cells, dysbiosis, neurotransmitters

## Abstract

The gastrointestinal (GI) tract of multicellular organisms, especially mammals, harbors a symbiotic commensal microbiota with diverse microorganisms including bacteria, fungi, viruses, and other microbial and eukaryotic species. This microbiota exerts an important role on intestinal function and contributes to host health. The microbiota, while benefiting from a nourishing environment, is involved in the development, metabolism and immunity of the host, contributing to the maintenance of homeostasis in the GI tract. The immune system orchestrates the maintenance of key features of host–microbe symbiosis via a unique immunological network that populates the intestinal wall with different immune cell populations. Intestinal epithelium contains lymphocytes in the intraepithelial (IEL) space between the tight junctions and the basal membrane of the gut epithelium. IELs are mostly CD8^+^ T cells, with the great majority of them expressing the CD8αα homodimer, and the γδ T cell receptor (TCR) instead of the αβ TCR expressed on conventional T cells. γδ T cells play a significant role in immune surveillance and tissue maintenance. This review provides an overview of how the microbiota regulates γδ T cells and the influence of microbiota-derived metabolites on γδ T cell responses, highlighting their impact on immune homeostasis. It also discusses intestinal neuro-immune regulation and how γδ T cells possess the ability to interact with both the microbiota and brain.

## 1. Introduction

The symbiotic commensal microbiota of mammals includes bacteria, fungi, viruses, and other microbial and eukaryotic species. These complex communities of microbes inhabit barrier surfaces of the digestive, respiratory, skin, and urogenital tracts and plays a crucial role in controlling many aspects of host physiology [1]. Despite having more than 100 trillion microbes in the intestinal tract, 90% of gut microbiota bacteria belong to two phyla, *Firmicutes and Bacteroidetes* [2,3], and one *Archaean* species, *Methanobrevibacter smithii* [4]. Humans are born germ-free, so our microbiota population is shaped by the external environment and nutrients. Although these microorganisms may be seen as foreign entities, our immune system has developed to foster a harmonious relationship with them. The gut microbiota assists in maintaining immune balance, resisting pathogens, and aiding in digestion, all in return for a nourishing environment. For example, probiotics are health-promoting microorganisms that work as antagonists by killing intestinal *Escherichia coli* and *Streptococcus* spp. by producing various metabolites and immunoregulators that enhance the body’s immune response and cytokine levels. Furthermore, *Lactobacillus* and *Bifidobacterium* spp. can enhance intestinal peristalsis, thereby reducing the retention of harmful or carcinogenic substances in the intestinal tract [5].

Gut microbes are believed to shape immunity and maintain homeostasis by interacting with the mucosal immune system through several highly integrated signaling systems and gene regulatory networks [6]. Viruses and fungi are prevalent within the gut and potentially hold significant functions in maintaining a healthy gut environment. Changes in the viral population within the gastrointestinal tract can impact the bacterial microbiome and its variety, as viruses can drive the evolution of bacterial resistance. For instance, viruses can facilitate the transfer of genetic material between bacterial communities horizontally, which can change the balance of different bacterial communities [7]. 

The immune system is a sophisticated and intricate collection of innate and adaptive components that work together to maintain the body’s homeostasis. A complex microbiota has occurred simultaneously with the development of distinct branches within the immune system, particularly those related to adaptive immunity. This suggests that the immune machinery has evolved to allow microbial communities to establish and maintain mutually beneficial relationships. In return, the microbiota actively supports and regulates the immune system response. Conditions affecting humans, such as allergies, autoimmune disorders, and inflammatory diseases, are often a consequence of a failure in controlling immune responses that target self-derived, microbiota-derived, or environmental antigens. Moreover, several factors such as dietary changes, the use of antibiotics, and the elimination of symbiotic partners have led to alterations in the composition and function of the microbiota [1]. Despite this close dependence, gut microbes and immune cells have their own niche within the gut: microbes are restricted to live in the gut lumen while immune cells are located in the gut tissue (epithelium and lamina propria). Commensal bacteria rarely break the intestinal barrier, and it is from the lumen that they participate in functions that are essential for host intestinal homeostasis. At the same time, host immune cells exert control on the microbiota population to prevent their invasion and systemic dissemination. Microbes that reach the gut mucosa interact with immune cells through proteins expressed on their surface or by releasing specific molecules, which triggers an immune response that results in microbial killing [8].

**Figure 1:**
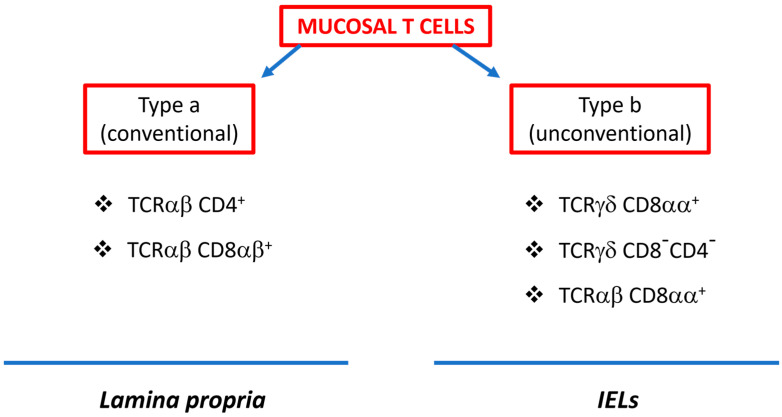
Classification of intestinal mucosal T cells.

Large repertoires of immune cell types populate the GI tract, making it the organ with the most complex immune system in the body, with variations in distribution and abundance. The intestinal epithelium predominantly accommodates T cells, while the lamina propria harbors a diverse range of immune cells, including B cells, T cells, innate lymphoid cells (ILCs), dendritic cells, macrophages, eosinophils, and mast cells [9]. These cells communicate with one another through cytokine production or cell–cell contact orchestrating the gut immune system [10]. Mucosal T cells are categorized into two major subsets based on their T cell receptor (TCR) and coreceptor expression. The first type is known as “type a” T cells, which express the conventional TCRαβ and coreceptors CD4 or CD8αβ. The second group are the non-conventional or “type b” mucosal T cells, which can express either TCRαβ or TCRγδ and typically include CD8αα homodimers. In general, the lamina propria predominantly houses “type a” T cells, while “type b” T cells are more prevalent in the mucosal epithelium [11] (Figure 1).

Aβ T cells and γδ T cells differ in their modes of antigen recognition. Aβ T cells specifically recognize antigen peptides presented by the major histocompatibility complex (MHC). However, γδ T cells lack this MHC restriction and utilize their γδTCR to recognize a broader range of structurally diverse and biologically unrelated substances, including lipopeptides, proteins derived from microorganisms, and self-proteins [12,13]. Therefore, γδ T cells can be classified as part of the innate immune system, where their TCRs function as pattern recognition receptors. However, γδ T cells can also participate in the adaptive immune response by rearranging TCR genes to generate diverse junctional combinations and developing a memory phenotype [14]. Thus, γδ T cells exhibit characteristics of both the adaptive and innate immune systems. 

This review summarizes the various mechanisms by which the crosstalk between gut microbiota and γδ T cells contributes to intestinal homeostasis, tolerance, and inflammation. Moreover, the microbiota–gut–brain axis is discussed along with the role of the microbiota in maintaining neuro-immune cells.

## 2. Microbiota and Intestinal Mucosa

The evolution of the immune system, especially the elements related to adaptive immunity, occurred alongside the emergence of a diverse microbiota. The gut-associated lymphoid tissue (GALT) in germ-free mice has decreased levels of immune cells in the lamina propria (T cells and IgA-producing B cells) and epithelial (intraepithelial lymphocytes; IELs) compartments [15,16,17], which highlights the importance of the gut microbiota in the development and maturation of the immune system. Moreover, gut microbiota can also regulate the secretion of antibacterial molecules by intestinal epithelial cells and IELs [18].

Commensal microorganisms colonize the gut after birth, and their antigens aid in the development of immunocompetency by stimulating lymphocyte proliferation in response to antigenic stimulation. Breast milk is a major contributor to the microbial colonization of the gut. Apart from its nutritional molecules, immune cells, and IgA antibodies [1], human breast milk also contains microorganisms, mainly bacteria belonging to the phyla *Firmicutes, Proteobacteria*, and *Actinobacteria* [19]. More than 800 bacterial species, mainly facultative anaerobic or strictly aerobic groups, with the great majority being *Bifidobacterium* spp., have been identified [20]. All components work together to influence the composition of the gut microbiota in infants and the way the host’s immune system responds to these bacteria. Maternal IgA antibodies help to restrict immune activation and bacterial attachment by binding to nutrients and bacterial substances. The presence of metabolites such as oligosaccharides, one of the most abundant molecules in breast milk, acts as prebiotic promoting the growth of predominantly beneficial bifidobacterial microbiota in the infant’s gastrointestinal system, modulating the gut microbiota, and preventing inflammation [1]. Cytokines produced by immune cells, on the other hand, are found in small amounts (picograms). Inflammatory cytokines, such as IL-1β, IL-6, IL-8, IL-12, TNFα, and IFNγ, are present in colostrum and mature breast milk. These cytokines can help to boost the immune system by stimulating the production of white blood cells and antibodies, while the immunosuppressive cytokine IL-10 helps to reduce inflammation and promote healing. Additionally, human milk bacteria have been reported to likely contribute to the infant’s gut microbiota colonization by influencing gut microbes through various mechanisms, including competition for nutrients and binding sites, direct inhibition, or participation in trophic chains [21]. 

The intestinal epithelial barrier (IEB) is considered a dynamic interface as it regulates the passage of nutrients, water, and electrolytes, while effectively limiting the passage of pathogens and toxins. IEB dysregulation can lead to increased intestinal permeability and thus facilitate the entrance of antigens that, subsequently, activate the immune system and initiate inflammatory responses [22]. The microbiota contributes to the proper functioning of the IEB by modulating the expression of tight junction molecules in the epithelial cells that control IEB permeability [23]. The microbiota has the potential to regulate host physiology through the production of a highly diverse metabolites repertoire, which play a critical role in the modulation of intestinal epithelial cells (IECs) and the maintenance of the gut epithelial barrier. Key among these metabolites are short-chain fatty acids (SCFAs), including acetic acid, butyric acid, and propionic acid, which are produced through bacterial fermentation of dietary fibers in the colon. These SCFAs penetrate the intestinal epithelial barrier and interact with host cells, influencing immune responses and disease risk. Moreover, SCFAs maintain the integrity of the intestinal epithelial barrier by regulating luminal pH and mucus production, providing fuel for epithelial cells, and impact the differentiation and proliferation of IECs [23,24]. It has been suggested that fermentable dietary fibers might facilitate mucin secretion by generating short-chain fatty acids like butyrate, the main short-chain fatty acid that plays a significant role in regulating mucin release. Butyrate serves as a major energy source for colonocytes and influences their gene expression through two mechanisms: inhibiting histone deacetylase (HDAC) or binding to G-protein coupled receptors (GPR41 or GPR43). On the other hand, acetate and propionate play roles beyond the intestine, acting as metabolic substrates for lipogenesis and gluconeogenesis [25]. Germ-free mice exhibit impaired mitochondrial respiration and increased autophagy in colonocytes compared to conventionally raised mice, indicating that the microbiota contributes to the survival of colonocytes [25].

**Figure 2:**
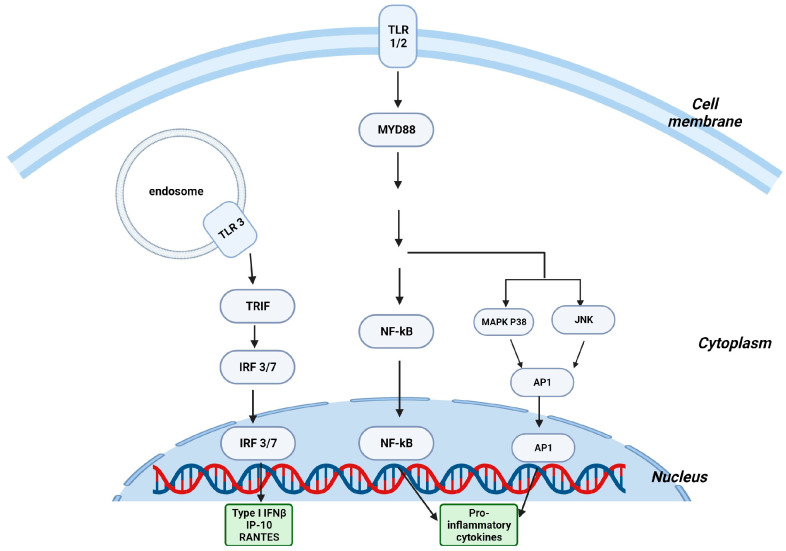
Microbiota regulates the immune response through the Toll-like receptor pathways. Toll-like receptor (TLR) pathways in γδ T cells. MyD88: myeloid differentiation primary response 88; NF-κB: nuclear factor kappa B; MAPK: mitogen-activated protein kinase; JNK: c-Jun N-terminal kinases; AP: activator protein 1; TRIF: TIR (Toll/interleukin-1 receptor) domain-containing adaptor protein inducing interferon beta; IRF: interferon-regulatory factor; IP-10: IFN-gamma-inducible protein 10; RANTES: regulated upon activation, normal T cell expressed and secreted, also known as CCL5. Created with BioRender.

The gastrointestinal tract, which is recognized as the largest interface for immune function in relation to the external environment, is constantly exposed to numerous substances that trigger immune responses. Considering the large number of antigens encountered by the host intestinal cells, it is crucial for the body to maintain a stable immune system within a healthy gut by maintaining a balance between its inflammatory reactions against harmful pathogens and its tolerance to beneficial bacteria and food antigens [26]. T cell homeostasis and differentiation are extensively modulated by gut bacteria. For example, Bacteroides fragilis and segmented filamentous bacteria (SFB) in the intestine have been shown to induce Tregs and Th17 cells, respectively, affecting the host’s response to infections. Moreover, signals originating from gut microbiota have the potential to regulate immune cells, enabling them to exhibit both pro-inflammatory and anti-inflammatory responses, thus affecting the susceptibility to diseases [27]. One of the primary mechanisms of communication between host and microbiota is through the recognition of conserved microbial-associated molecular patterns (MAMPs). These patterns are identified by pattern recognition receptors (PRRs) such as Toll-like receptors (TLRs) and C-type lectin receptors (CLRs), located on the cell membrane, and Nod-like receptors (NLRs) within the cytoplasm [28] and expressed on macrophages, monocytes, neutrophils, mast cells, dendritic cells (DCs), T lymphocytes and IECs [29]. Nevertheless, intestinal microorganisms express microbial-related molecular patterns that can activate TLRs on innate immune cells [30]. This activation triggers a series of intracellular signaling pathways that lead to the production of cytokines and chemokines and the transcription of different genes crucial for infection control. The predominant signaling pathways are the MyD88-dependent pathways that lead to the activation of transcription factor NF-κB and the mitogen-activated protein (MAP) kinases p38 and JNK, thus upregulating the expression of numerous proinflammatory cytokines. The second pathway is the TRIF-dependent pathway, activated by TLR3 and TLR4, which cause the activation of the transcription factors IRFs (interferon regulatory factors) 3 and 7. IRF3 and IRF7 will then induce the transcription of three genes, namely, (1) type I interferon-beta (IFNβ), (2) IFN-inducible protein 10 (IP-10/CXCL10), and (3) chemokine (C-C motif) ligand 5 (CCL5/RANTES), thus controlling infection by either recruiting more immune cells to the site of infection, stimulating the production of antimicrobial peptides (AMPs), or directly eliminating pathogens [31] (Figure 2).

A disruption in the balance of intestinal microorganisms leads to intestinal dysbacteriosis that can induce the differentiation of CD4^+^ and CD8^+^ T cells and, therefore, initiate an adaptive immune response [30]. The exact mechanism underlying this response is still under investigation, but recent studies have demonstrated that shifts in the metabolites released by microbial species can impact the function of immune cells. SCFAs are water-soluble, and therefore can be easily transported throughout the body; thus, they are believed to influence the differentiation, function, and regulation of T cells and other immune cells through promoting the release of pro- or anti-inflammatory cytokines required for specific effector functions [32]. Collectively, the above studies show that commensal microorganisms are essential for the development of the immune system. They contribute to the proper functioning of the IEB by the production of SCFAs and modulate the immune response through their MAMPs.

## 3. Intestinal **γδ** T Cells

Gamma delta (γδ) T cells were first discovered in 1984 by Saito and collaborators [33] and are a unique subset of T cells mainly present within the barrier tissues such as epithelia of skin, intestines, and lungs. Only a small percentage of γδ T cells are found in circulating blood and peripheral tissues [34]. Phenotypically, the intestinal γδ T cells are mostly CD8^+^ T cells [35], in contrast with the ones located in peripheral tissues that are CD4-CD8-[36]. γδ T cells can be classified in different subsets based on the type of γ- and δ-chains of the TCR. Herein, we concentrate on the γδ T cells present the intestinal epithelium, where the most abundant type expresses Vγ5 TCR in mice [37] and Vδ1Vγ2^+^ TCR in humans [38,39]. The main function of γδ T cells in the intestine is to maintain epithelial integrity and restrict the entrance of microbial pathogens and avoid their systemic dissemination, which makes them essential components of mucosal immunity. They are able to produce a wide range of cytokines, chemokines, perforins, and granzymes either constitutively or following interactions with surrounding cells like dendritic cells (DCs) and epithelial cells, or their products, as well as microbial metabolites in the intestinal lumen [40,41,42]. 

Lymphopoiesis of γδT cells occurs mainly in the thymus. Intestinal homing of γδ T cells is regulated by two molecules expressed on their cell membrane, the C-C chemokine receptor type 9 (CCR9) and the heterodimeric integrin αEβ7. CCR9 binds to its ligand CCL25, a chemokine that is abundantly produced by intestinal epithelial cells, to promote the intestinal migration of γδ T cells [43,44,45]. The integrin αE (CD103) forms a receptor complex with β7 that dimerizes and binds to the epithelial cell adhesion protein E-cadherin, expressed on intestinal epithelial cells, facilitating the entry and residence of γδ IELs in the intestinal epithelium [46,47].

Molecules such as IL-7, IL-15, butyrophilin-like molecules (BTNL), the ligand-activated transcription factor AhR (aryl hydrocarbon receptor), and aldo-keto reductase 1B8 (AKR1B8) are essential for the proliferation, survival, and maintenance of the homeostatic mechanism of intestinal γδ T cells [48]. The G protein-coupled receptor GPR18 can also regulate the expansion of γδ T cells in the gut and their positioning next to epithelial cells [49,50]. 

γδ T cells also express the natural killer group 2D receptor (NKG2D), an activating receptor associated with an adaptor molecule (DAP10) that enables signal transduction [51,52]. NKG2D receptors interact with their ligands, the MHC class I-related chains (MIC) A and B expressed on damaged cells [53]. Their binding during microbial infection provides a signal that activates the release of cytotoxic cytokines (IFNγ, TNFα) and, therefore, their effector cytolytic response [54]. This suggests that γδ cells are able to directly respond to infected or damaged cells expressing MIC. In this regard, it has been shown that human Vδ1 T cells carrying NKG2D receptors have the capability to kill MICA-positive tumor cells [52,55]. The γδTCR can also recognize ligands induced by infection or stress, like annexin A2 and endothelial protein C receptor (EPCR) [56,57]. Therefore, γδ IELs are able to recognize and respond to epithelial cell stress antigens or bacterial antigens expressed by infected cells [58]. Although γδ T cells display significant heterogeneity, and several distinct subsets have been identified based on the cytokine produced, there are two main functional subsets that are shared among multiple γδ T cell populations: (a) the IL-17-producing Th17-like subset and (b) the IFNγ-producing Th1-like subset [59]. 

IL-17 production is controlled by IL-23 and plays a vital role in coordinating innate immune functions. IL-17 facilitates the accumulation of neutrophils in peripheral tissues, aiding in pathogen clearance and host defense against various infections [60,61]. IL-17 also contributes to the preservation of mucosal barrier functions by promoting tight junction formation and mucin secretion. Similar to Th17 cells, IL-17-producing γδ T cells express CCR6, IL-23R, and AhR. Additionally, IL-17^+^ γδ T cells express TLR1, TLR2, and dectin-1. During infection, the binding of ligands to those pathogen-recognition receptors results in selective expansion and neutrophil recruitment [61]. CD30L/CD30 signaling pathways are crucial for the maintenance and activation of naturally occurring IL-17A-producing γδ T cells in mucosa-associated tissues and thus control the infection. Thus, mice deficient in CD30L or CD30 proteins were shown to be hypersusceptible to an acute infection by *Listeria monocytogenes*. At the early stages of *Listeria monocytogenes* infection, these mice have a higher bacterial load in the peritoneal cavity, coincident with a decrease in the number of neutrophils and IL-17A-producing γδ T cells [62].

In contrast, IFNγ-producing γδ T cells are crucial for an effective immune response against tumor development, viruses, intracellular bacteria, and protozoan parasites through enhancing phagocyte activity [63,64]. The production of IFNγ is greatly induced by IL-12 and IL-18, which are secreted by dendritic cells (DCs) [65]. Infection with a Plasmodium parasite activates both γδ T cells and DCs. Upon activation, γδ T cells express CD40L and produce IFNγ, which promote DC maturation and increase expression of MHC II molecules and co-stimulatory factors like CD86 on DCs, leading to an increase in IL-12 production by DCs. Consequently, the activated DCs induce the production of IFNγ from γδ T cells and the differentiation of naïve CD4^+^ T cells into Th1 cells to also produce IFNγ [64].

γδ T cells in the gut mucosa can also secrete other cytokines like IL-22 that, together with IL-17, can suppress microbial populations. IL-22 production is regulated by different factors, including IL-23R signaling, AhR, and RAR-related orphan receptor gamma (RORγt) [66,67]. γδ T cells can also produce growth factors such as keratinocyte growth factor (KGF) that helps to maintain the integrity of the epithelial barrier and stimulate the production of antimicrobial peptides (AMPs) by epithelial cells. KGF not only promotes the proliferation, maturation, and repair of damaged epithelial cells, but also regulates the formation of tight junctions and, therefore, intestinal permeability [68,69,70,71]. Moreover, γδ T cells in the intestinal epithelium can produce the antimicrobial peptide RegIIIγ (regenerating islet-derived protein 3) in response to bacterial infection of the epithelial cells. This antibacterial response is dependent on the stimulation of epithelial cell-intrinsic MyD88 signaling by bacteria, which suggests that epithelial cells provide microbe-dependent cues to γδ IELs [58]. 

Granzyme A and B are proteins highly expressed in γδT cells [72]. They are secreted constitutively at steady state as well as in response to CD103 ligation to the E-cadherin on epithelial cells facilitating the apoptotic cell death of the later ones [73]. Granzymes and perforins produced by γδ T cells are capable of killing infected and tumor cells [74]. γδ T cells can also produce immunosuppressant cytokines like TGF-β or IL-10 regulating other cells involved in the innate immunity response [75]. Table 1 summarizes the products secreted by γδ T cells in response to the different stimuli and the receptors involved in each case.

As mentioned above, γδ T cells are among the most abundant populations in the gut representing the first line of mucosal immune defense. However, there is variation in the frequency and function of these γδ T cells in the different layers of the gut [76]. IELs with up to 40% γδ T cells are intercalated between the epithelial cells and represent the first layer of intestinal T cells that support the epithelium barrier’s function, maintain microbiota symbiosis, and contribute to gastrointestinal inflammation and disease [77]. A second layer of intestinal γδ T cells is found among lamina propria lymphocytes (LPLs). These γδ LPLs are able to produce IL-17 and IL-22, which regulate the release of antimicrobial peptides and strengthen tight junctions between enterocytes to restrict bacterial dissemination and minimize intestinal inflammation. The third group of γδ T cells is located in the Peyer’s patches and likely plays a role in antigen presentation and promoting mucosal humoral immunity [76]. 

**Table 1:** The ligands involved in the modulation of intestinal γδ T cell functions.

Ligand	Receptor on γδ T Cells	Effector Factors	Function	References
IL-23	IL-23R	IL-22, IL-17	- Preservation of mucosal barrier function - Tight junction formation and secretion of AMP and mucins	[60,61,66,67]
Xenobiotics Natural products Microbiota metabolites Endogenous molecules	AhR (Aryl hydrocarbon receptor)			
CD30L	CD30	IL-17	- Preservation of mucosal barrier function - Antimicrobial	[62,78]
MICA/B (stress marker)	NKG2D (natural killer group2, member A)	IFNγ, TNFα KGF-1	- Fights against intracellular pathogens - Destruction of infected or damaged cells - Mucosal injury repair	[52,53,54,68,79]
MAMPs	TLR1 TLR2 Dectin-1	IFNγ IL-17 Reg III	- Expansion and neutrophil recruitment - Pathogen control	[58,61]
IL-12 IL-18	IL-12R IL-18R	IFNγ ↑CD40L	Enhancement of phagocytic activity	[64,65]
E-cadherin (on IECs)	αEβ7 integrin	Granzyme A/B Perforin	Lysis of infected and transformed intestinal cells	[72,80]
Microorganisms-derived proteins Lipopeptides Self-proteins Btnl	TCR	IFNγ IL-17 TNFα	Development of effector subsets	[39,56,57,58]
MIC (stress marker)	NKG2A	IL-10, TGFβ	- Treg expansion - Promotes integrity of the epithelium	[75]
IL-15	IL-15R		Maintenance, localization, proliferation, and maturation of γδT cells	[81]
Unknown	GPR18 (orphan G-coupled receptor)		Promotes entry, residence, and maturation of γδT cells in intestinal epithelium (homing)	[49]
CCL25 (epithelial cells)	CCR9		Promotes entry and residence of γδT cells in the intestinal epithelium (homing)	[43,44,45]

**Figure 3:**
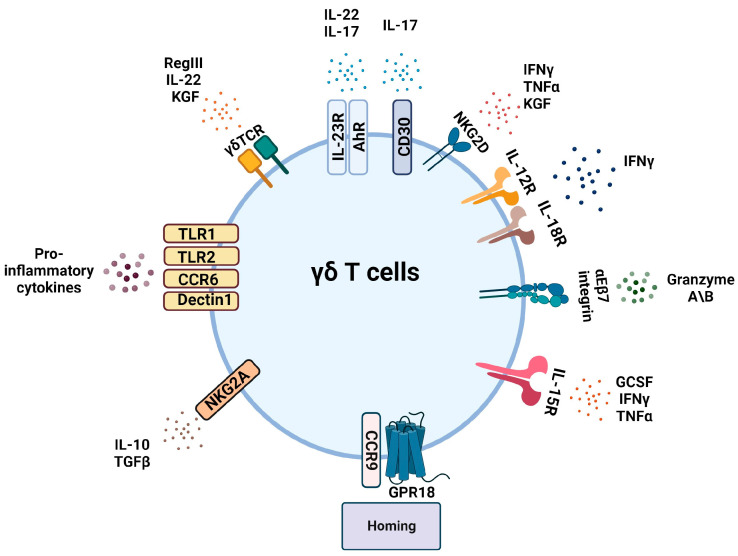
Plasticity of γδ T cells. γδ T cells can secrete a wide range of cytokines and chemokines in response to different ligands binding to their corresponding receptors. GPR18: G protein receptor 18; CCR9: C-C chemokine receptor type 9; NKG2A: natural killer group 2 member A; CCR6: C-C chemokine receptor type 6; TLR: toll-like receptor; γδTCR: γδ T cell receptor; IL: interleukin; AhR: aryl hydrocarbon receptor; CD30: also known as TNF receptor superfamily member 8; TGFβ: transforming growth factor β; RegIII: regenerating islet-derived protein 3; KGF: keratinocyte growth factor; IFNγ: interferon γ; TNFα: tumor necrosis factor α; GCSF: granulocytes-colony stimulating factor. Created with BioRender.

The special characteristics and great plasticity of the γδ T cells confer a crucial role in regulating the mucosal immune response against resident and invasive intestinal bacteria. Due to the quick response producing cytokines and growth factors in response to any changes, γδ T cells are major contributors to epithelial homeostasis (Figure 3).

## 4. Intestinal T Cells and Microbiota Interactions

γδ T cells in the intestinal epithelium are continuously interacting with the commensal gut microbiota that keeps them constitutively activated, as they are able to quickly respond to signals through their TCR, TLR, and NLR [82,83,84]. At the same time, γδ T cells contribute to the regulation of the microbial population and maintenance of intestinal homeostasis. So, it seems evident that there is a crosstalk between γδ T cells and gut microbiota. However, the exact mechanisms by which this interaction takes place have only recently begun to be understood. 

Early studies using germ-free (GF) mice reported that γδ T cells from the intraepithelial compartment were not affected by the intestinal microbiota [58,85]. However, a more recent study demonstrated that the gut microbiota is needed to maintain γδ T cells which, in turn, are essential for maintaining mucosal tolerance [86]. Using a broad-spectrum antibiotic treatment to deplete the microbiota, Rezende and collaborators reported a decrease in the number of γδ T cells, in both the lamina propria and intraepithelial compartment of the small intestine, as well as an enhancement in pro-inflammatory immune responses in treated mice. This suggests that the microbiota promotes oral tolerance by restraining inflammatory responses in the gut and by maintaining γδ T cell populations. Moreover, the same authors elegantly showed that γδ^−/−^ mice exhibited a dysregulated mucosal immune system characterized by increased Th17 and decreased Treg cells. This was associated with an impaired recruitment of tolerogenic DCs to the MLNs and a reduced capacity to produce IL-10 by CX3CR1^+^ mononuclear phagocytes. Interestingly, γδ^−/−^ mice also had marked intestinal dysbiosis affecting several bacterial species in the intestine. When the defective species were restored, the production of IL-10 increased, the Th17/Treg balance was normalized, and oral tolerance was rescued. The study identified *Ruminococcus gnavus* and *Akkermansia muciniphila* as essential microbes that interact with γδ T cells to maintain oral tolerance. Furthermore, γδ T cells secrete the micro-RNA let-7f that promotes the growth of *R. gnavus* to maintain homeostatic microbiota [86]. Similarly, two other studies demonstrated the importance of *Clostridium* spp. and *Bacteroides fragilis* in promoting the accumulation of IL-10^+^ Tregs in the colon, thus contributing to the maintenance of immune homeostasis in the intestine [87,88]. In contrast, SFB were shown to stimulate the production of IL-17 and IL-22, which promote the development of Th17 cells in mice [89]. 

Dupraz and collaborators demonstrated that SCFAs in the colon and cecum (mice and humans) exert a suppressive effect on IL-17-producing γδ T cells. Propionate directly impacts intestinal γδ T cells by inhibiting IL-17 and IL-22 production through the inhibition of HDAC [90]. However, several commensal microorganisms have been reported to have the capacity to increase the number of IL-17-producing γδ T cells (IL-17^+^IL-1R1^+^) through the VAV1 guanine nucleotide exchange factor [91]. Moreover, it has been shown that IL-17-producing γδ T cells protected mice against *C. difficile*-induced colitis [92]. Several studies have reported that phosphorylated microbial metabolites or phosphoantigens such as HMBPP (E-4-hydroxy-3-methyl-but-2-enyl-pyrophosphate), isopentenyl phosphate, aminobisphosphonate, and synthetic phosphoantigen derivatives are able to directly stimulate γδ T cells [40,93,94]. As mentioned earlier, γδ T cells can also directly recognize pathogen-associated molecular patterns through their TLRs and start an inflammatory response that will lead to the elimination of pathogens [61,91]. Microbial products or antigens could also act indirectly on γδ T cells through DCs via cell-to-cell interaction or cytokines [95].

Other microbiota-derived molecules have been shown to modulate the function of tissue-resident T cell populations through specific receptors or by entering the cells, to modulate the activity of specific transcription factors. Molecules such as SCFAs [96,97,98], secondary bile-acids [99], retinoic acid [100], dietary tryptophan derivatives [101,102], and polyamines [103] can induce, using different mechanisms, the differentiation of specific T cell populations and/or modulate their activity. Moreover, dietary tryptophan derivatives can function as AhR agonists. In this regard, *E. coli* and *Lactobacillus reuteri* have been reported to metabolize dietary products and convert them into ligands to the AhR and, subsequently, initiate an intracellular signal [101,102]. As a result, *E. coli* induced the release of proinflammatory cytokines in NKT cells [101], and *Lactobacillus reuteri* induced the differentiation of IELs [102]. More recently, it has been shown that the gut microbiota mediates the inhibition of lymphopoiesis which is associated with an increase in *Lactobacillus* and *Bacteroides*, and the production of butyrate under dietary restrictions (20–40% reduction in daily food intake) [104]. To date, no data have been reported specifically on γδ T cells. 

Enteric bacterial pathogens, such as *E. coli* and *Salmonella typhimurium*, are able to penetrate the intestinal barrier and induce the expression of RegIIIγ by γδ IELs through an MyD88-dependent manner [58]. The microbiota is also involved in the motility and positioning of γδ IELs within the intestinal epithelium, which is essential for an efficient surveillance of the epithelium [105]. Finally, microbial colonization of the GI tract has been shown to regulate the accessibility of enhancer regions of genes involved in a variety of signaling and metabolic pathways in IELs [106].

Collectively, these studies provide strong evidence to support the idea that microbiota can modulate immune responses in the GI tract by favoring the differentiation of specific T cell populations (Table 2).

## 5. Dysbiosis and Inflammation

Dysbiosis occurs as a result of an imbalance in the normal gut microbiota composition where the microbial diversity decreases, and harmful pro-inflammatory bacteria increase in number. During dysbiosis, microbiota is incapable of protecting against pathogenic organisms that induce inflammation and produce genotoxins or carcinogenic metabolites. Gastrointestinal infections or a short-term diet change can alter gut microbiota composition and cause dysbiosis. However, the gut microbiota is resilient and can revert to its original structure once physiological conditions return to normal. Nevertheless, in chronic diseases such as inflammatory bowel disease (IBD) or following exposure to broad-spectrum antibiotics, the gut microbiota can lose its resilience [25]. Changes in diet can result in inadequate nutrition of the microbiota contributing to compromised microbiota function and dysbiosis [107]. 

As described above, the mechanisms by which the microbiota controls intestinal homeostasis include substances like lipopolysaccharides, flagellins, and peptidoglycans that can influence cell survival, replication, apoptosis, and inflammation through direct interaction with intestinal cell receptors. Microbial dysbiosis disturbs the mucosal barrier, which can lead to the translocation of lipopolysaccharides (LPSs) and endotoxin accumulation, resulting in a hyperactive immune system [107,108]. Intestinal inflammatory and autoimmune diseases (colitis, IBD), as well as systemic autoimmune diseases such as type 1 diabetes, rheumatic arthritis, and multiple sclerosis, are driven by dysbiosis [108] in the intestinal microbiota and linked to an impaired epithelial barrier, inflammation, bacterial translocation, and a decline in Tregs in the gut mucosa. The imbalance in the ratio of helper T cells/Tregs plays a pivotal role in the development of inflammatory and autoimmune diseases. It has been shown that microbiota-derived signals are essential for the development and function of colonic Tregs (cTregs), which are critical for limiting intestinal inflammation [109]. A healthy microbiota contains anti-inflammatory bacteria like *Faecalibacterium prausnitzii* that induce the overexpression of the tight junction proteins and stimulate the differentiation of Tregs [110] or *Bacteroides fragilis* that produce the polysaccharide PSA and stimulate the production of IL-10 from T cells, limiting the activity of Th17 cells during intestinal inflammation [111]. However, other bacteria, like *Fusobacterium nucleatum*, promote inflammation by suppressing the activity of cytotoxic T cells and by altering the expression of microRNAs (miRNAs), resulting in the inhibition of autophagy [112]. 

IBD is an inflammatory gastrointestinal disease with different etiologies including the gut microbiota [113,114]. Microbiome populations of patients with IBD often exhibit a number of changes, not only in composition but also in diversity compared to that of healthy individuals [115,116]: the abundance of beneficial bacteria are reduced while harmful bacteria are increased [117]. Several mechanisms have been shown to be involved in the development of intestinal inflammation by the different intestinal microbial communities in IBD patients. Direct interaction between the microbiota and a variety of immune cells leads to the secretion of inflammatory factors. Many studies have shown that IBD is correlated with an increased activity of T cells secreting IL-17 and IL-22 cytokines [118] and a decrease in that of Tregs [119], which leads to an imbalance in the T17/Tregs ratio that mediates an exaggerated immune response, intestinal injury, and, therefore, the development and maintenance of IBD. Microbiota-derived metabolites, like SCFAs, tryptophan, and bile acids, can regulate the differentiation and expansion of Tregs, for which a decrease in these metabolites is related to an increase in inflammation [76]. All three types of metabolites have been reported to be decreased in IBD [120,121,122]. Another mechanism that contributes to intestinal inflammation in IBD is direct damage to the intestinal barrier due to physiological [123,124] or metabolic defects [125] of the epithelial cells that leads to an increased epithelial permeability and, therefore, inflammation.

Intestinal dysbiosis has also been associated with other inflammatory conditions in distal organs [126]. Several studies have shown that treatment with broad-spectrum antibiotics results in an impaired innate and adaptive immune response following systemic viral infections [127,128], which suggests the diffusion of microbial products and metabolites from the gut to the bloodstream [129]. Diseases such as chronic kidney disease [130] and systemic lupus erythematosus (extensively reviewed in [131]) have been associated with dysbiosis, which supports the important role that the microbiota plays in the development of chronic inflammatory diseases. Moreover, several anti-inflammatory molecules produced by intestinal bacteria have been identified, and they could be developed as effective treatments against chronic inflammatory diseases [132]. In summary, systemic immunity seems to be shaped by microbial products and any disturbance in these products will lead to defective host systemic immune responses and result in pathological inflammation [1].

## 6. Intestinal Neuro-Immune Regulation

The interaction between the nervous and immune systems has been investigated for a long time, but it was only in 2002 when the inflammatory reflex was described [133]. In this neuro-immune circuit, the afferent vagus nerve is stimulated by inflammatory molecules in the periphery and transmits this information to the brain. In response, the brain, through the efferent vagus nerve, suppresses the production of pro-inflammatory cytokines, especially TNFα. It is also known that neurons express membrane receptors for cytokines and other molecules produced by immune cells [134,135,136,137], as well as pattern recognition receptors that allow them to directly respond to microbial signals [138,139,140,141]. On the other hand, immune cells are not only able to respond to neurotransmitters and neuropeptides through their receptors, but some of them also possess the capacity to produce and metabolize neurotransmitters [138,139,142,143]. The gastrointestinal tract is controlled by a complex network of nerves, including the intrinsic enteric nervous system (ENS) and the extrinsic sympathetic, parasympathetic, and visceral afferent neurons [144]. We have evidence that intestinal γδ T cells express receptors for acetylcholine (ACh), allowing these cells to respond directly to cholinergic stimulation (unpublished observations). Furthermore, many nerves come into close contact with immune cells in the gastrointestinal mucosa, forming neuron–immune cell units that can be altered by signals from the gut lumen, such as nutrients or microbes [141,145,146,147]. For example, in the colonic lamina propria, myeloid cells and Tregs are located in the proximity of neuronal projections [148,149,150]. These myeloid cells express high levels of muscarinic acetylcholine (mACh) receptors that, upon ACh stimulation, upregulate the synthesis of retinoid acid [150]. In the absence of cholinergic stimulation (vagotomy), a reduction in the number of Tregs was observed, which suggests that the cholinergic pathway modulates Treg differentiation [150,151]. However, this modulation was disturbed in germ-free mice, indicating that microbial metabolites are required for this neuronal modulation of Tregs [150]. Moreover, the microbiota can modulate the number of enteric neurons and their projections to the lamina propria, as was shown in germ-free mice colonized with *Clostridium ramosum* [148]. Therefore, the gastrointestinal immune system and nervous system are able to constantly monitor the intestinal environment and rapidly respond to any threats. For example, our group demonstrated that cholinergic stimulation via systemic administration of acetylcholinesterase (AChE) inhibitors enhanced the gastrointestinal barrier’s defense mechanisms, leading to host protection against oral pathogens [152].

Despite the long-standing recognition of the importance of the gut–brain axis in maintaining homeostasis, our understanding of how distinct microbiota and their products regulate gut–brain function has become clear only over the past 10 years. There is evidence that microbes and the brain communicate through a variety of channels, including the immune system, tryptophan metabolism, vagus nerve, and enteric nervous system, using microbial metabolites, such as SCFAs, branched chain amino acids, and peptidoglycans. Several neuromodulatory metabolites produced by microbiota, including tryptophan precursors and metabolites, 5-hydroxytryptamine (5-HT), GABA, and catecholamines have been identified [153]. It was demonstrated that the metabolite 4-ethylphenylsulfate produced by gut bacteria can induce anxiety-like behavior in mice [154,155,156]. Moreover, the gut microbiota modulates locomotor activity in Drosophila, probably through bacterial-derived metabolites [157,158]. On the other hand, chronic psychosocial stress was shown to alter the composition of the gut microbiota, specifically decreasing the relative abundance of *Bacteroides* species (harmless bacteria) and increasing the relative abundance of *Clostridium* species (harmful bacteria). This change in microbiota composition was correlated with increased levels of proinflammatory cytokines, such as IL-6, and the chemokine CCL2 [159]. Moreover, chronic stress can disrupt the intestinal barrier, making it leaky and increasing the risk of bacterial translocation and thus the circulating levels of immunomodulatory bacterial cell wall components such as lipopolysaccharide [160] (Table 3).

SCFAs are among the most abundant metabolites of the gut microbiota and play a critical role in communication between the microbiota and the immune system. Over the past few years, a rich body of evidence has accumulated demonstrating how the three main SCFAs (namely acetate, propionate, and butyrate) do not only contribute to the development and effector properties of immune cells of the GI tract but also function as immunoregulatory mediators in a variety of autoimmune diseases. These include multiple sclerosis, type 1 diabetes, IBD, celiac disease, and rheumatoid arthritis. Generally, SCFAs act to downregulate proinflammatory responses, including IL-1β, IL-6, IL-17, and Th17 cells and increase IL-10 and Treg cell effector function, thus promoting an immunoregulatory environment (refer to [161] for a recent review on this topic). The role of propionate in multiple sclerosis has been well studied [162] (Table 3). In fact, in a landmark clinical study using propionate to supplement standard therapy in multiple sclerosis patients, a significant and sustained increase in functionally competent Treg cells, and a significant decrease in Th1 and Th17 cells, was observed after a mere 2 weeks of treatment [163]. These alterations were associated with marked improvement in disease severity, including a reduced annual relapse rate, disability stabilization, and reduced brain atrophy after 3 years of propionate supplementation [163]. In the context of γδ T cells, propionate has been shown to repress IL-17-producing mouse intestinal γδ T cells as well as the production of IL-17 by human IL-17-producing γδT cells from patients with IBD [91]. In another landmark study, a reduction in specific *Lactobacillus* species was associated with an increase in colonic IL-17-producing γδ T cells (γδ 17 T cells) and increased vulnerability to chronic social stress, leading to depression. Interestingly, these stress-susceptible cellular and behavioral phenotypes were shown to be causally mediated by dectin-1, an innate immune receptor expressed on γδ T cells [164]. These studies highlight the central role of intestinal γδ T cells in the gut–immune–brain axis.

Diabetes in mice and humans is associated with significant intestinal dysbiosis [165,166] and elevated circulating levels of branched-chain amino acids (BCAAs) synthesized by gut microbiota [167], specifically *Clostridiales* and *Lachnospiraceae* [166] (Table 3). In diabetes, there is also a reduction in vagal activity, manifested by decreased ACh levels and increased AChE [168], that is associated with a downregulation of tight junction proteins and an increased intestinal permeability. Yan and colleagues showed that treatment of diabetic mice with pyridostigmine (a reversible AChE inhibitor) resulted in an enhancement of vagal activity, restoration of homeostatic gut microbiota, decreased BCAA-producing microbiota and BCAA circulating levels, and improvement in intestinal permeability [166]. Given the involvement of γδ T cells in the maintenance of the epithelial barrier and the fact that γδ T cells express receptors for ACh, it is possible that these cells are part of the mechanism involved in this process. However further studies are needed to understand how the different T cell populations in the gut are regulated by neurotransmitters and other neuronal signals, and if this regulation is direct or indirect via antigen-presenting cells or soluble cytokine mediators. Moreover, future work is required to determine how the microbiota modulates these neuro-immune interactions and identify the specific microbiome and microbial products involved.

Parkinson’s disease (PD) is a neurodegenerative disease characterized by a progressive loss of dopaminergic neurons and accumulation of Lewis bodies in the central nervous system (CNS). Before the onset of the motor symptoms that characterize PD, patients develop GI symptoms related to damage in the integrity of the mucosal barriers [169,170] which led to severe inflammation and GI dysfunction. In fact, higher levels of TNF and other pro-inflammatory cytokines were found in PD patients when compared to control groups [171]. Moreover, α-synuclein (cytoskeleton protein that forms the Lewis bodies) has been reported to also accumulate in the neurons of the ENS and gut epithelium [172] which contribute to the degeneration of the gut tissue [173]. Dysbiosis has also been observed in PD patients, which contributes to chronic inflammation. Several studies have identified correlations between specific bacterial changes and PD severity. For instance, an increase in *Enterobacteriaceae* correlates with postural instability and gait difficulty, while decreased *Prevotellaceae* levels are associated with increased gut permeability and vitamin deficiencies [174]. Furthermore, reductions in the anti-inflammatory *Lactobacillaceae* family are likely involved in PD-related gut [174,175]. Lower levels of *Prevotellaceae*, *Lactobacillaceae*, and *Lachnospiraceae* have been linked to decreased SCFA [176,177] and ghrelin production, both critical for neuroprotection. Dysbiosis has been suggested as the cause of the accumulation of α-synuclein in the gut [178], which regulates the gut immune functions [179] such as recruitment of neutrophils and monocytes to the GI [180] and the maturation of DCs to produce IL-1β and IL-6 [180,181]. Gut inflammation would also lead to dysfunction of the mucosal barrier and bacterial dissemination, inducing neuroinflammation and neurodegeneration (Table 3). However, the exact mechanism is unclear and needs to be investigated further. Several immune cells have been found to reside in the meninges [182], where they secrete cytokines that interact with neurons, astrocytes, or microglia [183]. Among these immune cells are γδ T cells that regulate memory and some behaviors via the release of cytokines [184,185]. In neurodegenerative diseases, including PD, there is an inflammation of the CNS that is associated with the severity of the disease [186]. Moreover, immune reactions could be associated with the initiation and development of neurodegenerative diseases [187]. Although there is no conclusive evidence, γδ T cells could be among the first immune cells to arrive to the CNS and be in charge of regulating neuroinflammation [59]. PD patients exhibited a higher proportion of γδ T cells in cerebrospinal fluid (CSF) [188], but fewer γδ T cells circulating in their blood compared to healthy individuals [189], suggesting a potential migration of these cells from the bloodstream to the brain. Moreover, γδ T cells in the CSF were more activated than the ones in the blood [188]. Brain γδ T cells could be stimulated by microglia, the brain-resident macrophages, to produce IL-17. Microglia can be stimulated through the TLRs (2, 4, 7, and 9) and secrete the cytokines IL-1β and IL-23 that, in turn, will activate γδ T cells to produce IL-17 [190]. γδ T cells can also be activated directly by α-synuclein binding to their TLR2 [191]. In summary, γδ T cells can play essential and diverse roles in the initiation and progression of PD and other neurodegenerative diseases that should be investigated further.

**Table 3:** Gut dysbiosis-associated diseases.

Disease	Gut Microbiota	Microbiota Metabolites	Effect	References
Parkinson’s	↑ *Enterobacteriaceae*		Postural instability	
	↓ *Prevotellaceae* ↓ *Lactobacillaceae* ↓ *Lachnospiraceae*	↓ SCFA ↓ Ghrelin	↑ α-synuclein ↑ Inflammation ↑ Gut permeability ↑ Bacterial dissemination Neurodegeneration	[174,175,176,177,178,179,180]
Chronic Psychosocial Stress	↓ *Bacteroide* sps. ↑ *Clostridium* sps.		↑ IL-6 ↑ CCL2 ↑ Gut permeability	[159]
Multiple Sclerosis (MS)	↓ Propionate-generating bacteria	↓ Propionate	↑ IL-1β, IL-6, IL-17 ↑ IL-17 ↓ IL-10 ↓ Treg	[161,162]
Type 1 Diabetes	↑ *Clostridiales* order ↑ *Lachnospiraceae*	↑ BCAA	↑ Gut permeability	[165,166,167]

## 7. Conclusions

The interaction between the gut microbiota and the host’s immune system leads to diverse cellular and molecular responses that maintain the body’s homeostasis. The microbiota communicates with the mucosal immune system through different signaling pathways and gene regulatory networks that regulate immune cells and maintain the integrity of the intestinal epithelial barrier, restricting the entrance of pathogens and preventing chronic inflammation. Moreover, dysbiosis can impair the immune system and induce inflammation, immune sensitization, compromised barrier function, and autoimmune diseases. The effector functions of γδ T cells, which are essential components of the mucosal immune system, are modulated by microbial- and host-derived molecules, although the mechanisms are not completely understood yet. 

γδ T cells are involved in maintaining the homeostasis of the gut epithelial barrier and immune surveillance. Due to their inherent plasticity, these cells respond differentially to various antigens and are, therefore, versatile players in the regulation of immune responses in inflammatory diseases as well as in the defense against infections and tumors. In response to infections, γδ T cells can rapidly respond to avoid systemic dissemination and induce an adaptive immune response by recruiting neutrophils, macrophages, and DC. Despite the advancement in our understanding of γδ T cells as key regulators of mucosal physiology and pathology, there are several questions that remain unanswered. How do intestinal γδ T cells respond to neurotransmitters? Can neurotransmitters regulate their response to pathogens? Would this affect the microbiota composition? Another question remaining to be answered are whether γδ T cells can transmit signals to neurons and how. Neuroimmune regulatory networks are beginning to be dissected at the cellular and molecular levels. However, the full impact of the intestinal microbiota on these networks remains to be elucidated. Better understanding of these intricate relationships and their implications may contribute to the development of novel and effective therapeutic approaches for conditions related to metabolic diseases, dysbiosis, or cancer.

## Figures and Tables

**Table 2:** Modulation of T cells in the intestinal mucosa by microbiota and microbial metabolites.

Gut Microbiota	Target Cells	Effect	References
*Ruminococcus gnavus Akermansia muciniphilaas*	γδ T cells	↑ Oral tolerance	[86]
Phylum *Firmicutes* aerobic *Bacteroidetes*	γδ T cells	↑ IL-17	[91]
SFB (segmented filamentous bacteria)	γδ T cells	↑ IL-17 ↑ IL-22	[89]
*E. coli* *Salmonella typhymurium*	γδ T cells	↑ Reg III	[58]
Commensal microbiota	γδ T cells	↑ Mobility in the intestinal epithelium	[105]
Microbial metabolites (SCFAs, Propionate)	γδ T cells	↓ IL-17 ↓ IL-22	[90]
Phosphorylated microbial metabolites	γδ T cells	↑ Cell activity	[40,93,94]
*Clostridium* spp. *Bacteroid fragilis*	Tregs	↓ IL-10	[87,88]
*E. coli*	NK T cells	↑ Pro-inflammatory cytokines	[101]
Microbial metabolites (SCFAs, retinoid acid, polyamines, tryptophan derivatives)	T cells	↑ T cell differentiation ↑ T cell activity	[96,97,98,99,100,101,102,103,104,105]
Commensal microbiota	IELs	↑Chromatin accessibility	[106]
*Lactobacillus reuteri*	IELs	↑ T cell differentiation	[102]
*Lactobacillus*, *Bacteroides*	IELs	↑ Lymphopoiesis	[104]

## Data Availability

Not applicable.

## Abbreviations

ACh	acetycholine
AChE	acetylcholinesterase
AhR	aryl hydrocarbon receptor
AKR1B8	aldo-keto reductase 1B8
AMPs	antimicrobial peptides
AP	activator protein 1
BCAAs	branched-chain amino acids
BTNL	butyrophilin-like molecules
CCL	C-C chemokine ligand
CCL5	C-C chemokine ligand 5 also known as RANTES
CCR	C-C chemokine receptor
CD30	also known as TNF receptor superfamily member 8
CLR	C-type lectin receptors
CNS	central nervous system
CSF	cerebrospinal fluid
cTreg	colonic regulatory T cells
CXCL10	C-X-C motif chemokine ligand 10 or Interferon gamma-induced protein
CX3CR1	CX3C motif chemokine receptor 1 or G-protein coupled receptor 13
DAP10	DNAX-activating protein of 10KDa
DCs	dendritic cells
ENS	enteric nervous system
EPRC	endothelial protein C receptor
GABA	γ-Aminobutyric acid
GALT	gut-associated lymphoid tissue
GCSF	granulocytes-colony stimulating factor
GI	gastro-intestinal
GF	germ-free
GMPs	granulocyte–monocyte progenitors
GPR	G-protein coupled receptor
HDAC	histone deacetylase
HMBPP	4-hydroxy-3-methyl-but-2-enyl pyrophosphate
5-HT	5-hydroxytryptamine
IBD	inflammatory bowel disease
IEB	intestinal epithelial barrier
IEC	intestinal epithelial cells
IELs	intra-epithelial lymphocytes
IFNβ	interferon beta
IFNγ	interferon gamma
IL	interleukin
ILCs	innate lymphoid cells
IL23R	interleukin 23 receptor
IP-10	interferon-inducible protein 10
IRF	interferon regulatory factors
JNK	c-Jun N-terminal kinases
KGF	keratinocyte growth factor
LPLs	lamina propria lymphocytes
LPS	lipopolysaccharide
MAMPs	microbial-associated molecular patterns
MAPK	mitogen-activated protein kinase
MHC	major histocompatibility complex
MIC	MHC class-I- related chain
MNPs	mononuclear phagocytes
MyD88	myeloid differentiation primary response 88
miRNA	microRNA
NF-κB:	nuclear factor kappa B
NKG2D	natural killer group 2D receptor
NLR	Nod-like receptor
PD	Parkinson’s disease
PRR	patterns recognition receptor
RANTES	regulated upon activation, normal T cell expressed and secreted (CCL5)
RegIII	regenerating islet-derived protein 3
RORγ	RAR-related orphan receptor gamma
SCFA	short-chain fatty acids
SFB	segmented filamentous bacteria
TCR	T cell receptor
TGFβ	transforming growth factor beta
TNFα	tumor necrosis factor alpha
TLR	toll-like receptor
TRIF TIR	(Toll/interleukin-1 receptor) domain-containing adaptor protein inducing interferon beta
Treg	regulatory T cell
Th17	T helper 17-secreating cells
